# Nutrition Habits of Polish Nurses: An Approach

**DOI:** 10.3390/healthcare9070786

**Published:** 2021-06-22

**Authors:** Lucyna Gieniusz-Wojczyk, Józefa Dąbek, Halina Kulik

**Affiliations:** 1 Department of Propaedeutics of Nursing, School of Health Sciences in Katowice, Medical University of Silesia in Katowice, 20/24 Francuska Street, 40-027 Katowice, Poland; hbkulik@gmail.com; 2 Department of Cardiology, School of Health Sciences in Katowice, Medical University of Silesia in Katowice, 45/47 Ziołowa Street, 40-635 Katowice, Poland; jdabek@sum.edu.pl

**Keywords:** nurse, eating habits, workplace, health, diet, lifestyle

## Abstract

Background: Chronic stress, unlimited working time and the shift working system as well as sleep deficiency may lead to the occurrence of nutrition disorders among nurses. Aim: The aim of the paper was an assessment of the nutrition habits of nurses. It was an observational study conducted from June 2017 to May 2018 among nurses (*n* = 1080) in Silesia in Poland. Data was obtained using a range of questionnaires. Results: Body mass index (BMI) of the analysed nurses demonstrated overweight/obesity in 490 (45.5%) of them. Nearly all the diets of the analysed nurses (*n* = 1021; 94.5%) required improvement. Younger nurses (<31 years old) demonstrated a greater tendency to indulge in habitual overeating, and those having additional employment demonstrated poorer nutrition habits compared to those without additional employment. Conclusion: The analysed nurses made numerous dietary mistakes which, as a consequence, can lead to obesity. Emotional overeating was the dominant nutrition habit in the studied group of nurses. Nurses who were overweight/obese, nurses who had additional employment and younger nurses demonstrated tendencies toward improper nutrition choices, i.e., the tendency to overeat or restrain oneself from eating.

## 1. Introduction

In light of the increasing demand for caregiving and the increase in the average age of nursing staff [1] it is necessary to consider the introduction of a strategy for the improvement of their health. Undoubtedly, health complications among nurses affect the quality of patient care and the patient’s safety [2]. The promotion of a healthy lifestyle also helps nurses become better health educators and gives a better example to patients [3,4].

Health behaviours associated with nutrition are very important for the health of an individual. Earlier studies have demonstrated that poor nutrition choices among nurses are associated with professional challenges, including high mental burden [5]. Experiencing stress can encourage eating sweeter and fattier foods as well as snacking between meals [6,7]. Food high in energy suppresses the reaction to stress and additionally intensifies the urge to consume such food [8]. Shift work is a feature which causes additional increased stress [9]. Studies show that poor nutrition choices among nurses are associated with long working hours and shift work [10,11]. Other studies, in turn, indicate that bad quality sleep contributes to irregular meals and not observing diets [12] as well an increase in appetite [11] and, consequently, higher levels of excess weight and obesity [13]. In recent years, nutrition disorders have constituted an increasing, important social research and clinical problem. Despite numerous studies referring to the nutrition habits of nurses [5,14,15,16,17], there is a lack of research referring to poor nutrition habits, i.e., the tendency to overeat or to restrain oneself from eating which are important psychological factors in the etiopathogenesis of obesity, anorexia and bulimia among nurses. The aim of the paper was the assessment of nutrition habits of nurses in Poland.

## 2. Materials and Methods

### 2.1. Design and Participants

The present research was an observational, multicentre study conducted from June 2017 to May 2018 in the following healthcare units and training centres for nurses in Silesia: the Regional Specialised Hospital No. 4 in Bytom; Kornel Gibiński University Clinical Centre, Medical University of Silesia in Katowice; the Centre for Training Nurses and Midwives in Łagiewniki and the Centre of Postgraduate Education for Nurses and Midwives in Krosno. The study included 1064 nurses on the basis of the estimation of the size of the population of nurses in Poland (*n* = 288,395) (Main Chamber of Nurses and Midwives—NIPiP, 2017) (fraction size = 0.5; confidence level = 95%; maximum error = 3%).

Before handing out the questionnaires, the researcher informed all nurses about the aim and nature of the studies, about the application of the obtained results, and ensured anonymity. In addition, there was an initial instruction in each questionnaire in which the above-mentioned information was contained, and the questionnaires were distributed in envelopes. All the participants knowingly expressed consent for participation in the study. The study included nurses who had a diploma with the professional title of nurse. Due to the fact that professional work may be a factor influencing nutrition habits, the study included nurses with a longer job seniority, i.e., nurses who had been performing the profession for at least a year. The following nurses were excluded from the study: nurses who were pregnant, nurses who had less than a year of professional experience and nurses who declined consent for participation in the study. The exclusion was made on the basis of the questions contained in the introductory part of the questionnaire.

The Bioethics Committee of the Medical University of Silesia in Katowice approved the study (KNW 0022/KB/89/1/17). All the participants expressed consent to be included in the study by returning filled-in, anonymous questionnaires.

### 2.2. Gathering Data and the Research Tools

A total of 1200 questionnaires were distributed. After completing the questionnaire, the respondents personally returned them to the researcher (the response rate was 90%).

The survey included eight questions referring to demographic data. It also included the My Eating Habits (MEH) questionnaire by Nina Ogińska-Bulik and Leszek Putyński [18]. This questionnaire consists of 30 statements. Every diagnostic answer receives 1 point. The total number of points determines the general tendency toward improper nutrition choices (a high result obtained in the questionnaire proves an inclination for poor nutrition decisions, i.e., the tendency to overeat or restrain oneself from eating). According to the nature of overeating, three factors were identified, of which each comprised 10 questions: habitual overeating (0–10 points), emotional overeating (0–10 points) and the tendency to restraint from eating (0–10 points). According to the authors, this tool allows for diagnosing nutrition disorders, foreseeing the susceptibility to body mass increases [18]. Qualitative Assessment of Menu according to Z. Bielińska (Bielińska’s test) was applied for diet the assessment [19]. The mentioned scale assessed the method of nutrition understood as the frequency and regularity of eating meals and the frequency of using food products which were the source of particular nutrients. The assessment was performed on the basis of the awarded points; the higher the result, the better the nutrition method. A score of at least 31 indicates having an appropriate diet whereas obtaining a lower score is an indication of required improvement in diet [19].

### 2.3. Statistical Analysis

In the statistical elaboration, the Mann–Whitney U test was used for the assessment of the significance of differences between two groups in quantitative variables. A one-way ANOVA was applied for the assessment of differences between at least three groups in quantitative variables. The Kruskal–Wallis ANOVA test was performed for the comparison of more than two groups [20]. All statistical tests were calculated at the level of significance, alpha ≤ 0.05, and were performed using SPSS (Armonk, NY, USA) and Statistica (Hamburg, Germany).

## 3. Results

### 3.1. Characteristics of Studied Group

In total, the study comprised 1080 nurses aged 24–63 (mean age: 42.8). Nearly half of them (44%) had been working in their profession for more than 20 years *(n* = 484), and nearly 40% (*n* = 397) had additional employment. 45.5% of the nurses (*n* = 490) were overweight or obese (Table 1). A proper body mass was maintained by the highest number of the analysed nurses in the age range of up to 30, whereas in the age range of over 51, overweight/obesity dominated. Taking into consideration the point score assessment of menus according to Z. Bielińska (Qualitative Assessment of Menu), nearly all the menus of the analysed nurses (*n* = 1021; 94.5%), required improvement (Table 1).

The general characteristics of the studied group are summarised in Table 1.

### 3.2. Nutrition Habits 

The assessment of the nutrition disorders of nurses was performed using the My Eating Habits questionnaire [18]. Younger nurses (<31 years old) demonstrated a greater tendency for habitual overeating, in comparison to nurses older than 30 (*p* = 0.002, Kruskal–Wallis ANOVA). Moreover, nurses with additional employment demonstrated poorer nutrition habits (mean [standard deviation] score of 11.08 [5.98] on the MEH scale) in comparison to the studied subjects without additional employment (10.04 [5.93]; Z = −1.949; *p* = 0.05, Mann–Whitney U test) especially in the scope of emotional overeating (Z = −2.314; *p* = 0.021, Mann–Whitney U test; Table 2).

Moreover, overweight and obese nurses demonstrated a greater tendency toward inappropriate nutrition choices, especially with regards to emotional overeating in comparison to the remaining analysed subjects (*p* = 0.000, One-way ANOVA; Table 2).

The characteristics of the method of nutrition choices of the analysed nurses based on the point score assessment of the menu according to Z. Bielińska (Qualitative Assessment of Menu) is presented in Table 3. The analysed respondents made numerous mistakes in the selection of products. They received the highest number of zero points in the category referring to gaps between meals, and subsequently: the number of meals eaten containing animal protein and the frequency of eating brown bread and coarse groats (Table 3). 

In terms of the sociodemographic characteristics (age, marital status, additional employment, the type of ward and BMI) the analysed nurses did not significantly differ in the methods of nutrition (based on the point score assessment of the menu according to Z. Bielińska’s Qualitative Assessment of Menu). 

## 4. Discussion

The collection behaviours associated with nutrition are very important for the health of an individual. These behaviours may prevent or minimise the risk of the occurrence of hypercholesterolaemia, arterial hypertension, being overweight or the risk of obesity [21,22]. This seems to be especially significant in the aspect of the ageing nursing community and its challenges with medical facilities [1]. The fact that it is necessary to focus attention on conducting health prophylaxis among nurses is also proven by the results of multicentre studies indicating that, despite the possessed knowledge in the scope of health behaviours, including appropriate nutrition, nurses do not transfer it to their own habits and lifestyle [23,24].

Indeed, we have come to the conclusion that nearly all the nurses in our study would benefit from an improvement in their diets, similarly to other Polish studies [14,15,25]. 

Analysis of particular questions on the qualitative assessment of diet demonstrated that nearly half of the analysed nurses ate fewer than four meals per day and had excessive gaps between meals. According to the recommendations of the Food and Nutrition Institute (Poland) it is necessary to consume at least 4–5 meals per day while maintaining equal (3–4 h) gaps between them. Longer gaps between meals cause significant depletion of the body’s energy reserves, the symptoms of which include fatigue, weariness and lassitude as well as reduced ability to concentrate. Improper habits may lead to excessive energy supply and the reduction of the organism’s energy expenditure and, in consequence, increasing the risk of becoming overweight and obese [26]. In a similar percentage (50%; 49%) an appropriate number of meals was consumed by nurses analysed in the studies by B. Sińska et al. and B. Jankowska-Polańska et al. [14,15]. The consumption of an insufficient number of meals by nurses, which, as a result, contributed to excessive gaps between meals, was also confirmed by studies by A. Kucharska et al. [27]. This study also demonstrated insufficient consumption of fresh fruit and vegetables as a source of minerals, vitamins, fibre and natural antioxidants. In three quarters of the analysed nurses, fruit and vegetables were present in fewer than three meals; slightly poorer consumption habits were demonstrated in another Polish study [15]. In the studies by L. Perry et al. [17], only 8% of nurses and midwives were consumption-compliant with Australian guidelines. Nevertheless, these two professional groups observed nutrition recommendations in a greater percentage compared to the population of Australia. Much better results were obtained in Brazilian studies in which 45.5% of nurses consumed 3–4 portions of fruit and vegetables per day and 28.8%, more than five portions [16]. Besides fruit and vegetables, a valuable source of fibre in the daily diet is brown bread, coarse groats and wholemeal products which should be a component of the majority of meals [26]. These products were present in 84% of the analysed menus. An appropriate diet should also include an adequate amount of milk products that are rich in calcium and are a source of protein and vitamins: B1, B2, B6, B12, folic acid, vitamin A and magnesium. The appropriate intake of the discussed milk products in the diet reduces the risk of the development of insulin resistance and of metabolic syndrome [26]. In studies, 10% of the analysed nurses did not consume the aforementioned products. One of the assumptions of proper nutrition is also the supply of complete protein (eggs, fish, poultry) at least in three main meals during the day, whereas only 11% of the one-day menus subjected to qualitative analysis included animal protein in 3–4 meals during the day, and as much as 27% of menus got a zero score in this scope. 

The nutrition habits of an individual frequently take a compensatory form, satisfying the individual’s psychological needs, which leads to excessive eating and the development of obesity [28]. In our cohort, nearly half (45.5%) of nurses were overweight or obese, which reflects the percentage of adult women in Poland as a whole (40% in 2017) [29]. A similar percentage of overweight/obesity (44%) was observed earlier in Polish nurses [30]. Although this indicator of overweight/obesity is lower than that observed in other countries, including Australia (61%) [17], Scotland (69%) [31] and the USA (49%) [32], it is still worth reducing it. One of the reasons for excessive eating may be stress or negative emotions [33], and, as is commonly known, the work of nurses is very stressful [9,34,35,36,37]. The experienced discomfort associated with this causes the desire to reduce it. For some people, a way to cope with tension is by eating, despite the lack of hunger (emotional eating). In the study, this habit was significantly apparent in nurses with additional employment. Moreover, other studies demonstrate the risky effects of nurses’ work above standard time, in the form of consumption of alcohol [38]. Another reason for the excessive increase in body mass is the automatic consumption of food while performing various activities, e.g., during reading, working with a computer or watching television (habitual overeating). In this study, such eating was demonstrated by younger nurses. In the case of both emotional and habitual overeating, the quality and amount of food is not controlled which may have an impact on the application of nutrition restrictions in order to reduce body mass. These actions, in consequence, lead to the failure of self-control and to an increase in body mass [39].

In the case of emotional eating and habit overeating, the analysed nurses demonstrated 3–5 negative behaviours from the list of 10 possible behaviours, and in the whole MEH scale, in turn, it was on average 11 behaviours out of 30 possible. The studies by Ogińska-Bulik and Putyński [18] demonstrated that in comparison to women with normal body mass, the overweight women demonstrated a greater tendency toward emotional and habitual overeating and to more frequent restrictive behaviours in the diet. A similar tendency was demonstrated in the studies, which provides a guideline that hospital managers and occupational physicians should pay special attention to the assessment of the occurrence of nutrition disorders. The results obtained in this study provide guidelines regarding the aspects that need to be focused on in the medical care provided to medical staff in order to prevent obesity and, as a consequence, reduce employee absences caused by chronic diseases. 

Our study has some limitations. The data collected in this study were based on voluntary surveys conducted in one region of Poland. Therefore, it may not reflect all the nurses working in all the regions of country. However, we have taken into consideration a large cohort, which provides additional assurance that the data is representative for the national population of nurses in Poland. The study included using measurement tools based on self-description, which is associated with the possibility of the occurrence of variable social approval, i.e., the willingness of the analysed subjects to present themselves as better than they are in reality. Moreover, the study was cross-sectional in nature. Therefore, cause and effect relationships cannot be unambiguously determined on their basis.

## 5. Conclusions 

The studied nurses made numerous poor dietary decisions. Nearly all the nurses in study would benefit from an improvement in their diets. Emotional overeating was the dominating eating habit in the studied group of nurses. Nurses who were overweight/obese and nurses who had additional employment demonstrated inappropriate eating habits. Younger nurses should be made aware of the results of habitual overeating. An element which seems necessary is the introduction of diagnostics referring to nutrition disorders and pro-health nutrition education among nurses in order to minimise the diseases occurring in this group, which are associated with an irregular and stressful mode of work.

## Figures and Tables

**Table 1:** General characteristics of the analysed group of nurses in Poland.

Characteristic	No./Total No. (%)
Sex	
Male	28/1080 (2.6%)
Female	1052/1080 (97.4%)
Age (years)	
≤30	132/1080 (12.3%)
31–40	208/1080 (19.2%)
41–50	587/1080 (54.3%)
≥51	153/1080 (14.2%)
Marital status	
Single	760/1069 (71.1%)
Married	105/1069 (9.8%)
Divorced	178/1069 (16.7%)
Widowed	26/1069 (2.4%)
Additional employment	
Yes	397/1054 (36.8%)
No	638/1054 (63.2%)
Type of ward	
Surgical	354/1080 (32.7%)
Non-surgical	726/1080 (67.3%)
Body mass index	
<18.5 (underweight)	19/1080 (1.8%)
18.5–24.99 (healthy)	571/1080 (52.9%)
25–29.99 (overweight)	363/1080 (33.6%)
≥30 (obese)	127/1080 (11.8%)
Eating habits ^†^	
Normal diet	59/1080 (5.5%)
Poor diet	1021/1080 (94.5%)
Eating habits ^§^	M (SD)
Eating habits—total	10.65 (5.96)
Restraint from eating	3.56 (2.24)
Emotional overeating	4.10 (2.59)
Habit overeating	3.00 (2.62)

^†^ Based on Bielińska’s test for diet (Qualitative Assessment of Menu) ^§^ Based on the My Eating Habits (MEH) questionnaire.

**Table 2:** Characteristics of the analysed group of nurses, taking into consideration the nutrition habits and sociodemographic characteristics.

My Eating Habits ^§^	Age (Years)					
	≤30	31–40	41–50	≥51	Kruskal–Wallis ANOVA	
	M (SD)	M (SD)	M (SD)	M (SD)	*p*-Value	
Eating habits—total	10.80 (5.84)	10.90 (6.15)	10.53 (5.93)	10.65 (5.95)	0.803	
Restraint from eating	3.27 (2.27)	3.61 (2.30)	3.53 (2.24)	3.83 (2.16)	0.143	
Emotional overeating	4.05 (2.52)	4.08 (2.60)	4.12 (2.60)	4.08 (2.63)	0.993	
Habit overeating	3.48 (2.74)	3.21 (2.46)	2.87 (2.60)	2.75 (2.70)	0.002	
	**Marital Status**					
	**Single**	**Married**	**Divorced**	**Widowed**	**(One-way ANOVA)**	
	**M (SD)**	**M (SD)**	**M (SD)**	**M (SD)**	***p*-Value**	
Eating habits—total	10.61 (5.82)	10.71 (5.88)	10.76 (6.57)	9.85 (6.70)	0.904	
Restraint from eating	3.51 (2.30)	3.59 (2.24)	3.51 (2.35)	3.12 (1.66)	0.719	
Emotional overeating	3.89 (2.65)	4.18 (2.53)	4.12 (2.79)	3.88 (3.13)	0.589	
Habitual overeating	3.21 (2.68)	2.94 (2.56)	3.12 (2.75)	2.85 (3.12)	0.586	
	**Additional Employment**					
	**Yes**		**No**		**(Mann–Whitney U Test)**	
	**M (SD)**		**M (SD)**		***Z***	***p*-Value**
Eating habits—total	11.08 (5.98)		10.04 (5.93)		−1.949	0.050
Restraint from eating	3.70 (2.26)		3.47 (2.23)		−1.730	0.084
Emotional overeating	4.32 (2.52)		3.97 (2.63)		−2.314	0.021
Habitual overeating	3.06 (2.60)		2.96 (2.62)		−0.729	0.466
	**Type of Ward**					
	**Surgical**		**Non-Surgical**		**(Mann–Whitney U Test)**	
	**M (SD)**		**M (SD)**		***Z***	***p*-Value**
Eating habits—total	11.06 (6.09)		10.45 (5.89)		−1.546	0.122
Restraint from eating	3.61 (2.19)		3.53 (2.27)		−0.600	0.548
Emotional overeating	4.25 (2.59)		4.03 (2.59)		−1.458	0.145
Habitual overeating						
	**Body Mass Index (BMI)**					
	**Underweight (<18.5)**	**Healthy (18.5–24.99)**	**Overweight (25–29.99)**	**Obese (≥30)**	**(** **One-Way ANOVA** **)**	
	**M (SD)**	**M (SD)**	**M (SD)**	**M (SD)**	***p*-Value**	
Eating habits—total	6.16 (3.27)	9.13 (5.59)	12.26 (5.84)	13.60 (5.76)	0.0	
Restraint from eating	1.95 (1.72)	2.99 (2.06)	4.16 (2.18)	4.60 (2.44)	0.0	
Emotional overeating	1.95 (1.27)	3.46 (2.48)	4.82 (2.47)	5.24 (2.53)	0.0	
Habitual overeating	2.26 (2.31)	2.67 (2.43)	3.27 (2.74)	3.76 (2.84)	0.0	

Abbreviation: M (SD), mean (standard deviation). ^§^ Based on My Eating Habits (MEH) questionnaire.

**Table 3:** The characteristics of the method of nutrition of the analysed group of nurses based on the point score assessment of the menu according to Z. Bielińska’s Qualitative Assessment of Menu (*n* = 1080).

^†^ Nutrition Habits			
Assessed Characteristics	Answers	Score	No./Total No. (%)
The number of meals eaten	4–5 meals	5	512 (47.4%)
	3 meals	2	469 (43.4%)
	less than 3 meals	0	99 (9.2%)
Gaps between meals	more than 5 h	0	561 (51.9%)
	less than 5 h	5	519 (48.1%)
The presence of milk and milk products	2–3 meals	5	222 (20.6%)
	1 meal	2	749 (69.4%)
	none	0	109 (10.1%)
The presence of products providing animal protein	3–4 meals	5	120 (11.1%)
	2 meals	2	665 (61.6%)
	1 or in none	0	295 (27.3%)
The presence of fruit and vegetables	3–4 meals	5	259 (24.0%)
	2 meals	2	786 (72.8%)
	1 or in none	0	35 (3.2%)
The presence of fruit and vegetables rich in vitamin C and carotenes	3 meals	5	138 (12.8%)
	2 meals	2	867 (80.6%)
	in none	0	71 (6.6%)
The presence of raw salad	2 meals	5	135 (12.5%)
	1 meal	2	857 (79.4%)
	in none	0	88 (8.1%)
Brown bread or coarse groats	2 meals	5	276 (25.6%)
	1 meal	2	633 (58.6%)
	in none	0	171 (15.8%)

^†^ Based on Bielińska’s test for diet (Qualitative Assessment of Menu).

## Data Availability

Not applicable.
